# “We have to figure it out ourselves”: Transfeminine adolescents’ online sexual experiences and recommendations for supporting their sexual health and wellbeing

**DOI:** 10.3389/frph.2022.1034747

**Published:** 2023-01-16

**Authors:** Claire A. Coyne, Val Wongsomboon, Aaron K. Korpak, Kathryn Macapagal

**Affiliations:** ^1^ The Potocsnak Family Division of Adolescent and Young Adult Medicine, Ann & Robert H. Lurie Children’s Hospital, Chicago, IL, United States; ^2^ Department of Psychiatry and Behavioral Sciences, Northwestern University Feinberg School of Medicine, Chicago, IL, United States; ^3^ Institute for Sexual and Gender Minority Health and Wellbeing, Northwestern University, Chicago, IL, United States; ^4^ Department of Medical Social Sciences, Northwestern University Feinberg School of Medicine, Chicago, IL, United States

**Keywords:** adolescent sexual behavior, online partner seeking, social media, transgender, gender minority, transfeminine

## Abstract

The internet plays a significant role in adolescent sexual development. Sexual and gender minority (SGM) adolescents are more likely than their cisgender, heterosexual peers to use online spaces for sexual and romantic purposes, as they may have a smaller pool of potential partners and more concerns about the risks of in-person partner seeking. Among SGM adolescents, gender identity may shape how youth navigate online spaces for sexual purposes but there is limited research focused on transgender and gender diverse (TGD) adolescents’ online partner seeking. Previous research has focused on cisgender gay and bisexual boys’ experiences with sexual networking applications designed for adult men who have sex with men. This perspective article integrates clinical expertise and survey data from transfeminine adolescents (*N* = 21) in the United States reporting their online sexual behavior and experiences. We use qualitative data to describe the sexual health, safety, and wellbeing of transfeminine adolescents and offer suggestions for clinical assessment of online versus offline sexual activity and call for inclusive sexual health resources for transfeminine adolescents.

## Introduction

The following clinical scenario is a composite of several patients that reflects common issues that arise in the authors’ research and practice with transgender and gender diverse (TGD) adolescents.

*Ava is a 17-year-old white, affirmed transgender, pansexual girl who is engaged in psychotherapy to address symptoms of anxiety. Ava came out to friends and family as transgender at age 14, and has begun to socially transition (i.e., changed name, pronouns) at home and school. Her family and friends are affirming and supportive, and she plans to initiate gender affirming estrogen therapy. Ava is anxious in social situations and worries about being misgendered. She has a few close friends at school who are supportive and affirming, but reported these friends are all cisgender. She has friends in online spaces (i.e., Discord, Instagram), who primarily describe themselves as queer or transgender, but she also spends time talking to potential romantic and sexual partners in online spaces that are not exclusively for queer youth. In the context of medical appointments with her primary care physician and mental health provider, Ava reports that she is not sexually active. Ava has shared with her therapist that she is romantically involved with partners online and engages in sexual experiences with online partners. She described these experiences as complicated because she worries about when and how to disclose her identity and how to avoid transphobic responses from potential partners*.

TGD adolescents and young adults describe stigma associated with accessing medical and mental healthcare and report difficulty finding healthcare providers who are adequately trained to understand the healthcare needs of TGD individuals (1, 2). Healthcare providers may rely on TGD individuals to provide education about gender-related health issues, which exacerbates existing health care inequity for TGD youth relative to their cisgender peers. The American Psychological Association's practice guidelines for TGD individuals (3) encourage psychologists to discuss contraception and safe sex practices; however, there is limited information about whether existing sexual and reproductive health (SRH) resources, education or clinic-based counseling meets the needs of TGD youth (4, 5). Limited data on the early sexual experiences of TGD youth suggest higher risk of poor SRH outcomes (e.g., sexually transmitted infections, sexual assault) (6) and less sexual or romantic experience than cisgender peers (7). TGD youth may experience difficulty finding relevant, trustworthy SRH content for gender diverse individuals (4, 5) and due to prior stigmatizing experiences with healthcare providers, may avoid asking sexual health questions (5). Healthcare providers working with adolescents, though familiar with SRH resources and counseling, may lack training in sexual and gender minority health and familiarity with the specific needs of TGD youth.

Adolescents regardless of sexual orientation and gender identity seek romantic and sexual partners online (8). In the authors’ professional and research experience, online romantic and sexual partner seeking among sexual and gender minority adolescents is increasingly common, though there is scant documentation of their experiences in the literature. LGB adolescents have access to a smaller pool of potential partners in physical spaces (e.g., school, work) and may be hesitant to seek partners in person due to social stigma or not being out about their sexual orientation identity, which may render them more likely to seek partners online than heterosexual adolescents (8).

The limited research documenting LGBT adolescents’ experiences with online partner seeking has focused on gay, bisexual, and queer cisgender boys’ use of online sexual networking spaces for adults (e.g., 9, 10). Even less has documented the experiences of transgender and nonbinary adolescents (11). This initial work, conducted predominantly with transmasculine adolescents, has suggested that gender identity disclosure and online and offline safety with potential partners were particularly salient concerns in this sample. Similar concerns about identity disclosure and safety in online partnering have also been found among TGD young adults (12).

Transfeminine adolescents may have distinctly different experiences with online partner seeking both related to being TGD, and being feminine in particular, that may impact their sexual health, safety, and wellbeing in ways different from other sexual and gender minority youth. For example, it is well-documented that transfeminine adults experience disproportionately high rates of gender-based violence and sexual assault (13), and similar patterns may occur in adolescents. Also, transfeminine adolescents may be particularly vulnerable to coercion or power dynamics with older partners met online, among other experiences. That said, using the internet to seek partners may offer benefits, such as a safer way to explore and express their gender than doing so offline. To provide adequate and gender-inclusive SRH education and healthcare to transgender adolescents, it is critical to better understand their how they live their sexual and romantic lives both offline and online Drawing on preliminary data and observations from our research and clinical work with transfeminine adolescents, we offer suggestions for clinicians and educators working with TGD adolescents regarding sexual health discussions, inclusive education, and promotion of online sexual safety.

## Methods

Participants completed an online survey focused on online partner-seeking among transfeminine adolescents in the United States in spring 2022 (see Table 1 for demographics). The survey included closed- and open-ended questions regarding their romantic and/or sexual experiences with someone they first met online, such as motives for online partnering, best and worst experiences, gender identity disclosure, role of gender identity in online interactions, and incidence and types of online sexual activities. Our impressions and recommendations are based on data from 21 transgender girls and transfeminine adolescents (14–18 years old, *M* = 16.43), and the first author's clinical encounters with the same population in a pediatric gender clinic The survey was approved by the IRB.

**Table 1:** Sample demographics.

Variable	*M*	Range
Age	16.4	14–18
	*n*	%
Gender identity		
Trans woman	14	66.7
Transfeminine	7	33.3
Gender-identity outness		
Out to a selected few people	8	38.1
Out to most people	6	28.6
Out to everyone	6	28.6
Race		
Non-hispanic white	12	57.1
Non-hispanic black	3	14.3
Hispanic/Latinx	3	14.3
Biracial/Mutiracial	2	9.5
Native/Indigenous	1	4.8
Grade		
8th grade	1	4.8
10th grade	10	47.6
11th grade	4	19.0
12th grade	5	23.8
Not in school	1	4.8
Residence		
In an urban or city area	7	33.3
In a suburban area next to a city	10	47.6
In a small town or rural area	4	19.0
Social and medical transition interventions		
Changed name	15	71.4
Changed pronoun	16	76.2
Changed clothing	1	4.8
Used puberty blockers	2	9.5
Took hormones	2	9.5
Had voice therapy	2	9.5
Thought about having gender affirming surgery	15	71.4
Had gender affirming surgery	1	4.8
Relationship status		
Single	11	52.4
In a serious relationship	7	33.3
It's complicated	3	14.3

All participants are assigned male at birth. *N* = 21.

## Results

### Where and why do adolescents meet partners online?

All participants reported meeting their partners on social media or messaging platforms, such as Instagram, Snapchat, and Discord. Eight reported using mobile dating apps (e.g., Grindr) and, of those, six were under 18 years old. Sexual identity development and sexual exploration were common motivations for online partnering, as noted by one teen: “It was a great place to start seeing how I enjoy being intimate presenting as female, and overall it helped me learn a lot about myself” (trans woman, 18, Latinx). Participants also reported seeking a safe space or connection with like-minded others. One participant indicated: “I was in a safe space for trans women and other people questioning their gender and I formed a connection with someone” (trans woman, 17, White). Developing relationships with other transgender girls online can provide a connection to gender inclusive spaces that are not accessible to transgender adolescents like Ava in their offline life. At the same time, transfeminine adolescents like Ava may also seek out partners in online spaces not explicitly identified as gender inclusive or queer and need to develop skills to navigate these spaces (e.g., whether she discloses her gender identity and how to do that).

Participants indicated the difficulty they experienced finding acceptance from potential partners in offline spaces motivated their online partner-seeking. Many participants also mentioned loneliness, boredom, and need for acceptance as main motivators for online partnering, as one person noted: “I felt lonely and like I would never find someone that will accept and love me for who I truly am. I was desperate to find someone who would see me no different than any other cis girl” (trans woman, 16, Latinx). For transfeminine adolescents, who may not be able to transition socially or medically in offline spaces, online interactions may provide a valuable opportunity to practice the social scripts for sexual identity exploration in their affirmed gender, much like their cisgender peers. Given the potential social and emotional benefits of online partnering, healthcare providers should approach conversations about online partners with curiosity rather than stigmatizing how transfeminine adolescents use online spaces.

### What sexual behaviors do transfeminine adolescents engage in with online-met partners?

Online sexual activities with an online-met partner were reported by 16 (76%) of the participants. The types of online sexual activities endorsed included sexting or sex chats (*n* = 15, 94%), sending or receiving nudes or almost-nude pictures (*n* = 13, 87%), and engaging in webcam or phone sex with the partner they first met online (*n* = 10, 62%). Most (*n* = 11, 69%) reported masturbating during these online sexual activities and being sexually satisfied from their masturbation.

Overall, most participants said that their online sexual experience helped them explore their sexuality or learn about themselves as a sexual person. They also indicated that their online sexual experience played a role in establishing their sexual and/or gender identity. Although most participants engaged in online sexual activities, few (*n* = 5, 31%) engaged in physical, in-person sexual activities with their online-met partners. One person explicitly stated that they were only interested in “e-sex.” For some transfeminine adolescents, online sexual/romantic activities and relationships may be sufficient without needing to interact with their partners in offline settings. Anecdotally, some transfeminine adolescents describe fulfilling long-term online relationships they develop with partners met in online communities. Consequently, healthcare providers’ recommendations about healthy relationships and safe sex practices need to address challenges specific to online spaces. For a minor like Ava, who has not engaged in sexual behaviors in-person, recommendations about healthy relationships and sexual health need to consider risks specific to online spaces (e.g., legal issues for nude photo sharing) and concerns relevant to her gender identity (e.g., asking partners to use correct language for her body parts while sexting).

### What are transfeminine adolescents’ experiences with disclosing TGD identity to prospective online partners?

In navigating online spaces, transfeminine adolescents described positive experiences that affirm their gender identity (e.g., encouraging responses from partners after disclosing their identity) but also negative expectations associated with gender-related discrimination and rejection that contribute to fear and anxiety about their gender identity. Many survey participants disclosed their gender identity early in order to preempt negative reactions from transphobic individuals, as one person noted: “…[S]o they don't judge me automatically and make assumptions, sometimes they don't care and we flirt more or date. Sometimes we unadd each other” (trans woman, 15, White). To help transfeminine adolescents effectively with decision-making about disclosure, healthcare providers need to understand online-specific concerns (e.g., online harassment, exploitation) and positive or negative consequences of choosing not to disclose gender identity with online-met partners.

When asked how gender identity played a role in how they interacted with online-met partners, survey participants said that identifying as a transgender woman or transfeminine made online spaces more dangerous for them. Participants felt the need to be more cautious when interacting with people online due to fear of harassment, discrimination, or online attacks. As a result, many participants described disclosing their gender identity as a proactive, protective strategy to mitigate harm.

*I’ve always told people I’ve met my identity because it isn’t really safe for most trans women since they get killed for just being them, which is really sad. When I talk to people online I always tell them before the conversation keeps going (trans woman, 16, Hispanic and Asian)*.

Many also had concerns related to passing (i.e., being perceived correctly as their affirmed gender identity) and how their partners might respond to shared photos or videos. However, participants still acknowledged that they were able to express their femininity online in ways that would not be possible offline, and that online relationships provided a layer of protection for them because they were not meeting partners in person.

*[An online space] changes how I wish to present myself as a transgender woman. It makes me want to present myself in a more feminine way that is traditionally harder to do in real life (trans woman, 16, White)*.

### What concerns do transfeminine adolescents have about online partnering?

Participants frequently discussed negative experiences and safety concerns during their interactions with online-met partners. Many adolescents mentioned concerns about not knowing who prospective partners really were and how to prove their identity when interacting online. They also reported instances of catfishing, harassment, objectification, fetishization, and exploitation. Notably, most of the negative interactions (e.g., harassment) mentioned by participants occurred with online partners who described themselves as cisgender men, as noted by one participant:

*“My worst experiences were mostly on Snapchat, only sexual and nonconsensual or barely consensual. They all involved cisgender men/boys. Usually they involve being sent nudes without asking or showing interest in them. Many were also transphobic.” (transfeminine, 16, White)*.

Perhaps unsurprisingly, almost all participants received no formal sexual health education or parental guidance advice regarding online dating or relationships. Consequently, decisions about how to engage with online-met partners or navigate online sexual relationships were guided by information gathered online, through social networks or trial and error (14).

*A lot of us are never told about this. We figure it out ourselves. This can lead to us being…manipulated a lot. Especially among lower income (trans woman, 16, White)*

Concerns about safety are not restricted to online partnering, however managing safety in online settings requires different strategies than partnering in-person. Participants wanted skills to navigate online partnering but found existing sexual health resources lack relevant information about TGD-specific safety risks (e.g., exploitation, fetishization of transgender women) or how to reduce harm:

*There’s a lot of emphasis on abstinence across the board in health education that isn’t helpful. We need to know how to engage in these things safely (transfeminine, 16, White)*

Exposure to online harassment or manipulation (e.g., catfishing or luring someone into a relationship using a fictional online persona) can reinforce stigmatizing, transphobic messages about transfeminine identities and contribute to emotional distress. Relevant SRH resources for transfeminine adolescents should address physical and emotional safety with special attention to how gender minority stigma and stressors impact romantic and sexual decision-making. For Ava, learning strategies for managing safety and responding to transphobic messages may also include emotion regulation skills for coping with distress.

## Discussion and recommendations

It is developmentally normal for adolescents to explore sexual and romantic relationships online. This may be especially true for sexual and gender minority adolescents, more particularly TGD adolescents, who may have concerns about the availability of in-person partners in their social networks and the safety of in person sexual and romantic interactions. Compared to our prior work (11) the findings suggest that TGD adolescents may meet partners in more varied online spaces than do cisgender gay and bisexual boys. This difference likely reflects the presence of sexual networking applications focused on cisgender men, and the relative absence of online spaces for sexual minority women and TGD individuals.

We found that gender identity, particularly transfeminine identity and feminine gender presentation, appears to amplify safety concerns. This is unsurprising given national survey data demonstrating transgender women experience physical and sexual assaults at rates nearly double their transgender male peers (15). Though not explicitly mentioned by participants, safety concerns also vary based on other minoritized identities (e.g., race/ethnicity). For racially and ethnically minoritized transfeminine adolescents, experiences with racism in online spaces can compound safety concerns. Indeed, transgender girls/transfeminine youth may have a heightened awareness of gender-based violence and are seeking relevant SRH resources to support safe, healthy sexual exploration online and in-person.

The perspectives of transfeminine adolescents point to several concrete actions that healthcare providers or educators can take when working with transfeminine adolescents. First, healthcare providers should ask about the role of online spaces and apps in adolescents’ sexual exploration, behavior, and partnering. Given the pervasive nature of the internet in contemporary adolescents’ lives, it is important to avoid catastrophizing or stigmatizing online sexual behavior. For transfeminine adolescents, there may be safe ways to establish developmentally appropriate, affirming romantic or sexual relationships online that would be difficult to establish in their in-person social networks. That said, it is important for health care providers to differentiate between normative sexual behaviors versus behavior that may be associated with poor SRH and mental health outcomes. For example, health care providers can provide education about consent in online spaces and information about sexting laws regarding the sharing of sexual images or nudity online that apply to individuals under the age of 18 using resources like the Cyberbullying Research Center (16).

Second, transfeminine adolescents may be engaging in romantic and sexual relationships only online, only in-person or in both settings with the same or different partners. Sexual health questions and SRH education need to include both health outcomes and risks associated with in-person (e.g., sexually transmitted infections) and/or online (e.g., legality and risks of photo sharing, risks associated with sharing personal identifiable information, sexual behavior with individuals they only know online). As such, Table 2 offers some example questions that providers might ask adolescents—regardless of sexual orientation or gender identity—to better understand the role of online spaces in their sexual lives.

**Table 2:** Sample questions for clinicians to ask adolescents about online sexual and relationship experiences.

*Normalize behavior and create safe space*
I’ve talked to many teens who use internet and social media to explore their sexuality, and even meet sexual partners. This includes dating and sex apps intended for adults over 18. I’d like to ask you some questions about your experiences to better understand what's going on in your life. No judgment here—the goal is to understand things that might affect your health and wellbeing, including your sexual health.
*Assess lifetime and current online sexual experiences*
• Have you ever used an app, website, or other online space to connect with sexual or romantic partners or to have sexual experiences? If so, what sorts of websites or apps have you used? • Are you currently using any websites or apps, and if so what are they? How often do you use them? • Have you ever met anyone in person who you initially met online?
*Assess motivation for online sexual behavior*
• Young people look for sex and partners online for many different reasons. What are yours? • What are you hoping to get out of exploring these websites/apps?
*Assess types of online relationships and sexual behavior*
• What kind of relationships are you interested in finding online? • Do you have a romantic partner in-person or online? If so, what are things you like about that relationship? • What are things that are challenging about that relationship? • What kinds of sex are you having online? What kinds of things do you do online for sexual reasons? (probe for sharing nudes/pics, sexual chatting, camming, etc)
*Assess perceived safety*
• What do you do—if anything—to protect your safety when talking with potential partners online? • What are behaviors or signs you look for to help determine if a partner is safe? • How do you discuss consent, boundaries, and what you like and don’t like with people you’re talking to online? • Have you ever felt unsafe or uncomfortable during sexual interactions with other people online? How did you deal with this?
*Assess access to sexual health resources and education*
• Where do you find information about sexual health and relationships online? • What information do you want but have not been able to find?
*Assess parental involvement/knowledge of online sexual behavior*
• Do your parent (s)/guardian (s) know you use the internet or apps for sexual reasons? • How do you think your parent (s)/guardian (s) would feel if they found out? What are the rules in your home about social media use?
*Invite questions and leave discussion open-ended*
I know this can be a sensitive topic to talk about, and I appreciate you sharing this with me. I am happy to talk with you about this any time. What questions do you have for me?

Finally, sexual health educators should include transfeminine and transgender women in the development of educational resources and materials for adolescents and healthcare providers. Incorporating the lived experiences and effective strategies used by transgender women in online and offline spaces will improve the utility of sexual health resources. Survey participants highlighted their informal reliance on transfeminine peers to navigate challenges around disclosure and safety. Community-based participatory research approaches can be used by sexual health educators to engage transfeminine adolescents and young adults in the collaborative development of acceptable and relevant resources.

## Conclusion

This perspective article presents clinical observations and preliminary data with a small sample of TGD adolescents. However, the patterns we have noticed across our experiences are consistent and point to the need for more empirical work with this population, which can guide medical and behavioral health practice and sexual health education inclusive of TGD adolescents. In addition, we only focused on transfeminine teens, but as online dating and sexual experiences likely differ by gender identity and gender presentation, future research should compare the online sexual experiences of nonbinary adolescents with those of transfeminine and transmasculine adolescents. Together, these efforts can shed light on how providers and educators can best support adolescents’ sexual development, autonomy, and safety.

## Data Availability

Data will be shared upon reasonable request.
